# Biological Variation in Peripheral Inflammation and Oxidative Stress Biomarkers in Individuals with Gaucher Disease

**DOI:** 10.3390/ijms23169189

**Published:** 2022-08-16

**Authors:** Siddhee A. Sahasrabudhe, Marcia R. Terluk, Kyle D. Rudser, James C. Cloyd, Reena V. Kartha

**Affiliations:** 1 Center for Orphan Drug Research, Department of Experimental and Clinical Pharmacology, College of Pharmacy, University of Minnesota, Minneapolis, MN 55455, USA; 2 Division of Biostatistics, School of Public Health, University of Minnesota, Minneapolis, MN 55454, USA

**Keywords:** Gaucher disease, oxidative stress, inflammation, biomarkers, variability, therapy, Bland–Altman

## Abstract

The lack of reliable biomarkers is a significant challenge impeding progress in orphan drug development. For appropriate interpretation of intervention-based results or for evaluating candidate biomarkers, other things being equal, lower variability in biomarker measurement would be helpful. However, variability in rare disease biomarkers is often poorly understood. Type 1 Gaucher disease (GD1) is one such rare lysosomal storage disorder. Oxidative stress and inflammation have been linked to the pathophysiology of GD1 and validated measures of these processes can provide predictive value for treatment success or disease progression. This study was undertaken to investigate and compare the extent of longitudinal biological variation over a three-month period for various blood-based oxidative stress and inflammation markers in participants with GD1 on stable standard-of-care therapy (N = 13), treatment-naïve participants with GD1 (N = 5), and in age- and gender-matched healthy volunteers (N = 18). We utilized Bland–Altman plots for visual comparison of the biological variability among the three measurements. We also report group-wise means and the percentage of coefficient of variation (%CV) for 15 biomarkers. Qualitatively, we show specific markers (IL-1Ra, IL-8, and MIP-1b) to be consistently altered in GD1, irrespective of therapy status, highlighting the need for adjunctive therapies that can target and modulate these biomarkers. This information can help guide the selection of candidate biomarkers for future intervention-based studies in GD1 patients.

## 1. Introduction

Type 1 Gaucher disease (GD1) is rare, yet the most common autosomal recessive lysosomal storage disorder [1]. It is caused by mutations in the *GBA1* gene leading to glucocerebrosidase (GCase) enzyme deficiency [2]. Additionally, there is evidence of misfolded GCase protein exacerbating the effects of accumulation of its substrates (glucosylceramide and glucosylsphingosine) [3,4]. An abnormal accumulation of glycosphingolipids within the lysosomes results in the appearance of pathological ‘Gaucher cells’, the hallmark sign of GD1, leading to hepatosplenomegaly [5,6,7,8]. Gaucher disease is a multisystem disorder that spans beyond the liver and spleen and includes immune and oxidative imbalances, contributing to the local and systemic manifestations. Involvement of inflammation and oxidative stress, either of primary or indirect importance in the complex manifestations of GD, has long been speculated, hypothesized, and studied [9,10,11,12].

In the United States, five treatments are FDA-approved for treating the non-neuronopathic manifestations of GD. These treatments augment the deficient enzyme through enzyme replacement (enzyme replacement therapy, ERT, e.g., imiglucerase, velaglucerase alfa, taliglucerase alfa) or relieve substrate accumulation by acting upstream to suppress the synthesis of substrates (substrate reduction therapy, SRT, e.g., miglustat and eliglustat) [13,14]. All of these treatments manage GD to varying extents and the results are often variable. While bone pain and chronic fatigue are the most debilitating problems for some patients [15], current treatments only partially address these issues, and the molecular mechanisms contributing to these symptoms remain poorly understood [16]. Many patients, despite being disease stable on one of the approved treatments, continue to experience symptoms related to pain and fatigue, potentially due to unresolved or partially resolved inflammation and oxidative stress [17].

Several molecular biomarkers have been explored in the clinical development of GD1 treatments. For instance, chitotriosidase (CHITO), glucosylceramide (GL1), angiotensin-converting enzyme (ACE), tartrate-resistant acid phosphatase (TRAP), chemokine ligand 18 (CCL18, previously known as pulmonary and activation-regulated chemokine PARC), macrophage inflammatory protein-1 beta (MIP-1b) etc. [18,19]. A recent report suggests that compared to a placebo, eliglustat treatment in treatment-naïve adults with GD 1 resulted in statistically significant improvements in organ volumes, hemoglobin concentration, and platelet count, with a commensurate decrease in inflammatory biomarkers [20,21]. However, these biomarkers were only of exploratory importance. Moreover, those explorations did not put forth disease targets beyond glucosylceramide synthase. Improvements in liver and spleen size were mostly the primary outcome measures in these pivotal studies. Although the current treatments are beneficial in improving these aspects of GD, hepatosplenomegaly-related evaluations fall short in acknowledging the multisystem nature of this disease. The glycosphingolipid accumulation and the release of pro-inflammation biomolecules by the affected macrophages can contribute to the pathophysiology and chronic manifestations of the disease such as pain and fatigue, and there is interest in exploring key players in these processes as potential biomarkers [22]. The Gaucher cells are mainly macrophages infiltrated into the bone, brain, visceral organs, and other tissues [23]. The abnormal macrophages can produce and release macrophage-derived factors (chemokines) and cytokines. An imbalance in the levels of reactive oxygen species (ROS) and antioxidants through the pathological accumulation of glycosphingolipids results in oxidative stress. Several biomarkers of oxidative stress have also been widely investigated. However, small population sizes, phenotypic differences, the cross-sectional nature of the studies with single measurements, assay differences, and a focus on specific biomarkers have made interpretation of the results difficult. 

Having a reasonable estimate of baseline values for inflammation and oxidative stress markers with some confidence in the repeatability of measurements that captures inherent biological variability is an important first step in evaluating the treatment effect in any intervention-based cohort study. In this study our objective was to estimate plausible baseline values of 15 biomarkers of interest along with the extent of the inherent variability, both intra-subject and inter-subject, observed in their repeated measurements. We also highlight the importance of adopting new adjunctive treatments such as antioxidants and/or anti-inflammatory agents to manage patients with GD. 

## 2. Results

### 2.1. Participant Demographics

A total of 36 participants were recruited for our study: 5 participants were treatment-naïve, 13 were stable on a GD1 therapy, and 18 were healthy controls. The GD-naïve participants in our study were distinctly older than the other two groups; however, due to the rarity of the treatment-naïve GD1 population, in an era with five approved treatments, our recruitment options for this study were limited (Table 1). The ratio of males to females in all three groups was approximately 1. All three groups were predominantly Caucasian. Clinical records for patients with GD1 were explored and measures of chitotriosidase (CHITO), angiotensin-converting enzyme (ACE), and tartrate-resistant acid phosphatase (TRAP) were retrospectively summarized. Literature-reported normal ranges in healthy individuals for CHITO, ACE, and TRAP are included in Table 1 [24]. It can be noted that in the GD-treated group, TRAP and ACE concentrations were within the normal healthy range. Although the CHITO concentrations were lower in the GD-treated group compared with the GD-naïve group, the values were higher than the normal levels. Seventeen participants had a known genotype: 4/5 (80%) of the GD-naïve and 5/13 (38.5%) of the GD-treated group were homozygous while the rest were compound heterozygotes. The most common second allele was L444P (p.L483P) (N = 5). There was a wide range of comedications used by the participants with GD to primarily treat symptoms such as pain, anxiety, depression, etc. 

### 2.2. Variability in Measures Related to Oxidative Stress

**Figure 1:**
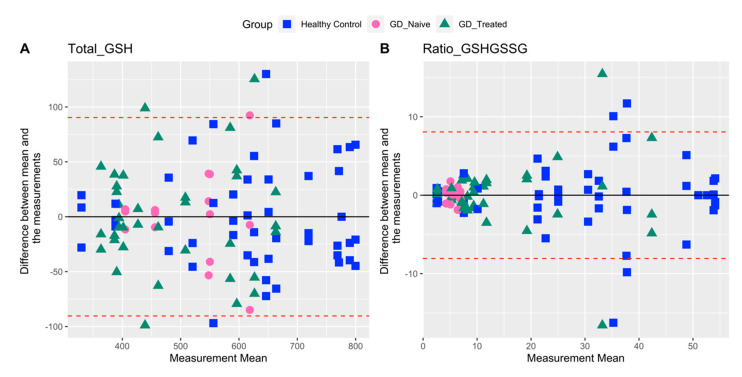
Bland–Altman plots for total GSH and GSH/GSSG comparing agreement between three repeat biomarker measurements for each individual. Upper and lower limits of agreement (dashed red lines) correspond to two standard deviations (SD) from the mean difference (solid black line).

Glutathione: For total GSH the data are largely distributed evenly throughout the measured range for all the three groups (Figure 1A). There is a pattern of increasing variability with increasing mean values in all three groups. In GD-naïve participants, the redox ratio was lower and more tightly clustered compared with the other two groups (Figure 1B). Overall, total GSH was less variable compared with the redox ratio, as evident in a distinctly higher %CV for the latter (Table 2).

**Figure 2:**
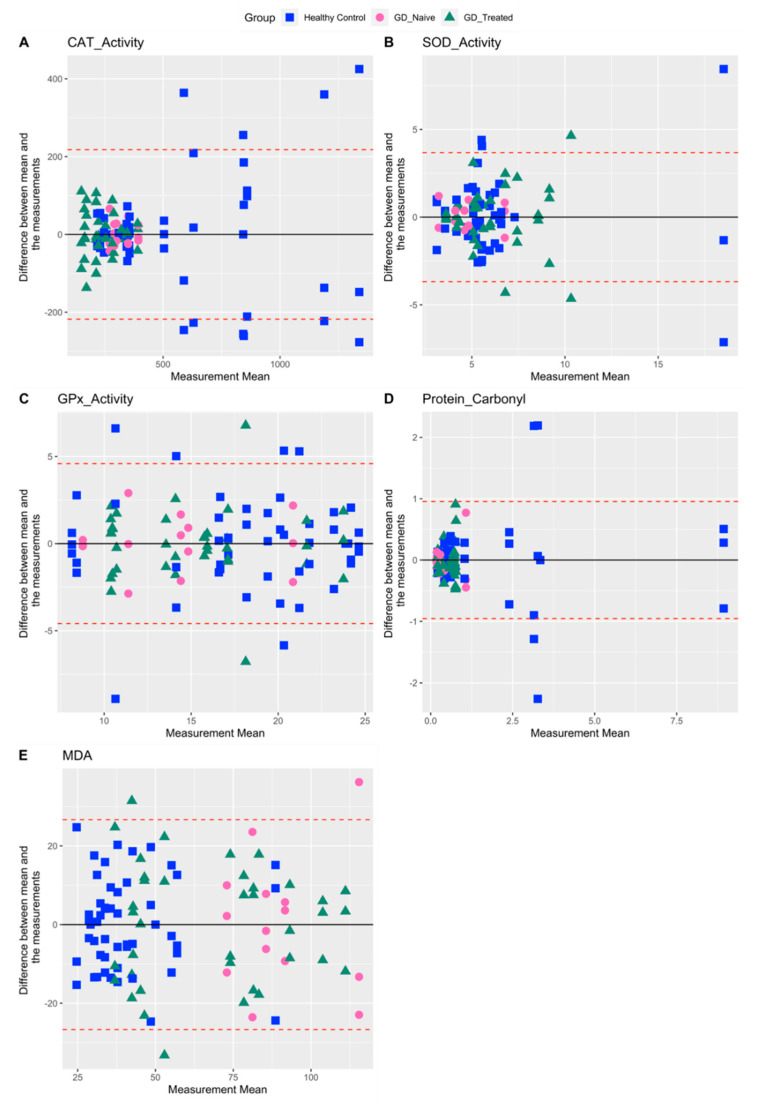
Bland–Altman plots for other oxidative stress related biomarkers comparing agreement between three repeat biomarker measurements for each individual. Upper and lower limits of agreement (dashed red lines) correspond to two standard deviations (SD) from the mean difference (solid black line).

Among other measures of oxidative stress including CAT, SOD, GPx activity, protein carbonyl, and MDA, protein carbonyl showed the highest %CV for healthy controls and GD-naïve individuals (143.9 and 104.7, respectively) (Table 2). Protein carbonyl values in the GD-treated group were more tightly clustered around the lower end of the measured range (Figure 2D). Except for GPx activity, where observations from all three groups overlapped (Figure 2C), we found that measurements in healthy volunteers were more variable in terms of the range they spanned and intra-subject values. (Table 1 and Figure 2A,B,D,E). In contrast, patients with GD showed lower values and less variability (both inter- and intra-subject), especially for the antioxidant CAT (Figure 2A), whereas the lipid peroxidation measure MDA showed the opposite trend (Figure 2E). 

### 2.3. Variability in Measures Related to Inflammation

**Figure 3:**
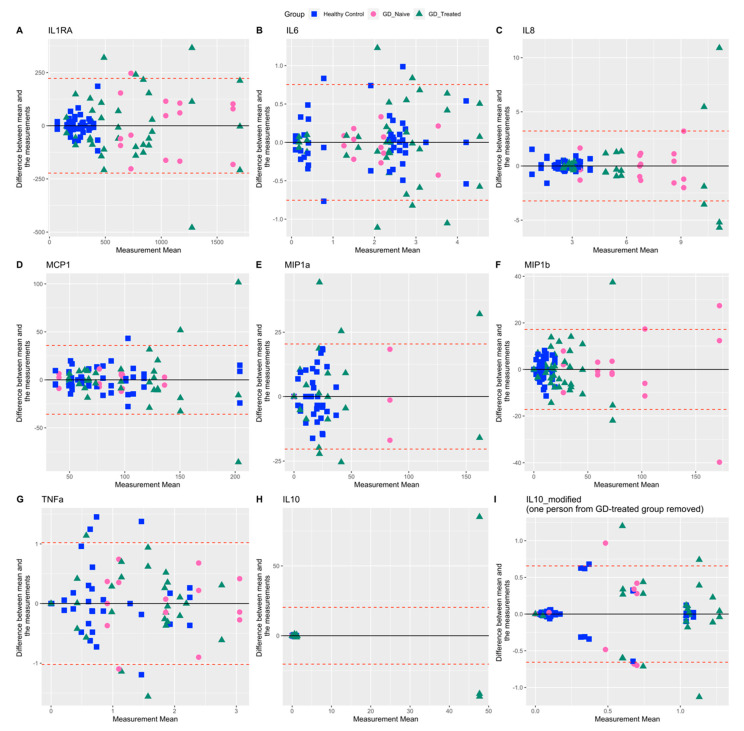
Bland–Altman plots for inflammation related biomarkers comparing agreement between three repeat biomarker measurements for each individual. Upper and lower limits of agreement (dashed red lines) correspond to two standard deviations (SD) from the mean difference (solid black line). IL-10_modified: Data for one participant from the GD-treated group was omitted because of apparent outlying measurements.

Among all the inflammation biomarkers, IL-6, MCP1, and TNFa showed a distinct random scatter of measurement differences along entire concentration ranges. Higher measurements of IL-8, MCP1, and MIP-1b showed more variable repeat values for the GD-treated group. Measurements of IL1RA, IL-8, MIP-1a, and MIP-1b were less variable in healthy controls compared with the other groups and were tightly clustered around the lower end of the measured range (Figure 3). IL-10 showed a very high %CV, owing largely to one person in the GD-treated group. We report summary statistics and %CV with and without data from that participant (Table 2, Table 3). Moreover, on visual inspection, inflammation markers IL-1Ra, IL-8, and MIP-1b showed distinct differences between healthy controls and patients with GD1, irrespective of treatment status (Figure 3A,C,F).

## 3. Discussion

To our knowledge, this is the first report summarizing the biological variation in oxidative stress and inflammation biomarkers in patients with GD1. We had previously reported significant differences in key oxidative stress biomarkers in participants with GD depending on their treatment status [17]. In GD-treated participants, the parameters of oxidative stress generally fell between the controls and the untreated, indicating partial resolution of oxidative stress following standard-of-care GD treatments. We concluded that underlying oxidative stress may contribute to GD1 pathophysiology and that the therapies targeting oxidative stress may prove useful as adjuvant treatments for GD [17]. Now we take that investigation one step closer to a prospective intervention-based clinical trial, and report our findings related to variability in the repeated measurements of oxidative stress and inflammation markers to help guide candidate biomarker selection. Generally, biomarkers with lower variability (e.g., total GSH, GPx activity) would be preferred over those with higher variance to support a more efficient evaluation of differences between groups. 

It has been proven that there is a relationship between the inflammation markers released and clinical manifestations in GD [25,26,27]. Multiple studies have confirmed that cytokine levels in GD1 patients are significantly higher than in control individuals [8,23,28,29,30,31]. In addition, MIP-1a, and MIP-1b are significantly elevated in the plasma of GD patients and are thought to mediate abnormally high bone reabsorption through osteoclasts activity [8,32]. Non-enzymatic and enzymatic antioxidant molecules, along with other oxidative stress markers such as plasma lipid peroxidation and protein oxidation, have been studied in patients with GD-1 [10,33,34,35]. These aberrant biomolecule findings have received increasing attention as evident in some of the ongoing clinical trials [36,37,38,39]. Although multiple reports suggest elevated inflammation and oxidative stress, coupled with impeded antioxidant protection in patients with GD1, the available information is not sufficient to guide future clinical trials as it does not inform researchers regarding the plausible biomarker measurement ranges and the variability around those measurements. Often, the variability observed in healthy populations or in non-rare diseases is extrapolated to make up for the lack of information; however, any deviations in those could substantially affect the statistical power. Variability can dramatically affect statistical power during hypothesis testing. It is thus essential to have a good understanding of the variability present in the biomarkers of interest measured in the population of interest and how it may impact our ability to draw conclusions. Even though we cannot limit or remove the variability, we can plan an appropriate target population size and outcome biomarkers to ensure that the study has adequate power. Our study is of value as it provides an estimate of the variability (standard deviation) in the three groups studied that can aid power and sample size estimates for future studies. 

We used a qualitative visual representation of the bias and agreement between the repeated biomarker measurements using a Bland–Altman analysis [40,41,42], which is a widely used method for its simplicity and highly informative graphical display. It is often used for assessing the level of agreement between two analytical methods. Although its application is in two measurements, we applied that to study agreement between three repeat measurements on the same participant, where the analytical methods employed were the same. We observed that the pair differences increased systematically with the mean for many of the biomarker measurements. In the figures, we also show the horizontal lines corresponding to 1.96 × SD. For most biomarkers, these bands are wide and capture much of the data; however, it is important to note that it does not necessarily have any clinical relevance. 

Cytokines and antioxidant levels are heterogeneous, even in healthy people. Some of the factors that contribute to inter-individual variability are genetics, age, sex, lifestyle, hygiene factors, microbiome etc. [43,44,45]. These markers are non-specific and display intra-individual differences with exercise [46,47,48] and diurnal rhythm [45,49,50,51,52]. Despite that, most of the previous reports that explored immunological profiles and oxidative imbalance in the GD1 patient population, were based on a single time point. The results could be biased because of the less than adequate sampling scheme utilized in those studies. To be able to evaluate the effect of experimental antioxidant treatment, we needed to ensure “true” baseline values. We needed minimize the number of repeated measurements due to cost, time, and other resources. In our case, the question of interest was to estimate the repeatability of measurements. Since data collected in more than two waves for baseline opens the possibility of using the mean or median of those measures for any future group-wise comparisons [53], our study design incorporated three baseline sample collections. 

There are a few limitations associated with our analysis. Firstly, only a few assays were performed in triplicate, and therefore, we were not able to obtain the corresponding analytical variation. However, we believe that its contribution to the overall variability observed would be minimal as the analytical variation of the assays performed using commercially available kits was determined previously by the manufacturers. In addition, the chromatography assays performed in-house were validated per the FDA’s analytical guidance [54] to ensure accuracy and precision. There were several missing samples (Table 3); however, given the small influence of missing samples on Bland–Altman visualizations, we retained data from all the measurements for all participants that were available. There is a range of high-throughput assays available commercially that are founded on fluorescent signal-enhancement principles (e.g., enzyme immunoassay, chemiluminescence, flow-cytometry) and ‘omics’ [55]. Studies in large population-based cohorts report significant variability across these methods [55]. The effect of sample handling processes (e.g., storage, freeze-thaw cycles, anticoagulants used) and sample preparation processes (e.g., matrix effect, dilution) might also impact the measurements [49]. Our estimates of repeatability and variability measurements correspond to the methods that we employed. Lastly, we report variability findings from a smaller patient cohort, compared with other studies focused on healthy volunteers [56] or those suffering from common diseases [57,58].

## 4. Materials and Methods

This study was conducted at two research sites and the study protocols were approved by the Human Research Protection Programs at the University of Minnesota (UMN) and New York University (NYU). The study was listed on ClinicalTrials.gov [NCT02437396 and NCT02583672]. All participants were adults and written informed consent was recorded for all prior to their enrollment in the study.

### 4.1. Study Participants

Eighteen US-based adult participants with genetically and/or metabolically confirmed diagnoses of GD1 were recruited from the UMN, NYU, and nationally with the assistance of the National Gaucher Foundation and study investigators. Appropriate age- and gender-matched healthy controls (N = 18) were recruited through the UMN Study Finder. The healthy controls were current non-smokers, without any known concurrent medical conditions to ensure the integrity of the data collected. All the participants of this study were enrolled between 2015 and 2018. The participants with GD1 who were on treatment (N = 13) were required to be on a specific ERT or SRT regimen for at least 2 years and needed to be dose stable for at least 6 months prior to their enrollment in the study. There were five treatment-naïve GD participants enrolled in this study. Other comedications were recorded and people who used antioxidants three weeks prior to the study were excluded.

### 4.2. Blood Sample Collection

Blood samples were collected from all participants at three time points, each one month apart, over a three-month period. The samples were processed to separate plasma and red blood cells (RBCs) following a standard lab protocol. The plasma and the RBCs were then aliquoted and frozen at −80 °C until further analysis. The blood samples were analyzed for the following oxidative stress related biomarkers:

(1) Intracellular glutathione (GSH) status measured as total GSH and the redox ratio of reduced/oxidized glutathione (GSH/GSSG) in RBCs;
(2) The activity of intracellular antioxidant enzymes-catalase (CAT), superoxide dismutase (SOD), and Glutathione peroxidase (GPx) in RBCs;
(3) Plasma lipid peroxidation profile as determined by malondialdehyde (MDA) levels;
(4) Oxidative modification of proteins determined as protein carbonylation (Protein carbonyl) levels in plasma.

Blood samples were tested for the following inflammation-related biomarkers:

(1) Pro-inflammation cytokines: interleukin-1 receptor antagonist (IL-1RA), interleukins (IL-6, IL-8, IL-10), tumor necrosis factor-alpha (TNFa), monocyte chemoattractant protein-1 (MCP-1) measured in plasma;
(2) Inflammation markers related to skeletal manifestations: macrophage inflammatory protein (MIP)-1alpha (MIP-1a) and MIP-1beta (MIP-1b), measured in plasma.

### 4.3. Measurement of Oxidative Stress

Catalase, SOD, GPx, MDA (measured as Thiobarbituric Acid Reactive Substances, TBARS), and protein carbonylation assays were measured using commercially available kits (Cayman Chemical, Ann Arbor, MI, USA) following the manufacturer’s instructions with minor modifications as previously described [17]. Total GSH and GSH redox status were measured using liquid chromatography-tandem mass spectrometry (LC-MS/MS) methods as detailed previously [17].

### 4.4. Measurement of Inflammation

The Luminex Performance Human Cytokine Panel A (R&D Systems, MN) was used to quantify a panel of plasma cytokines and chemokines such as IL1RA, IL-6, IL-8, MCP-1, MIP-1a, and MIP-1b, TNFa, and IL-10.

### 4.5. Statistical Analysis

Descriptive statistics were tabulated overall and by group (healthy control, GD-naïve, GD-treated), including the mean and standard deviation, interquartile range, and %CV for all biomarkers. Modified Bland–Altman plots were plotted by taking the mean of three measurements per participant and the difference between each measurement and the calculated mean (intra-participant difference) [40,41]. Horizontal lines were also plotted corresponding to the overall mean ± 1.96 × (SD) where SD represents the standard deviation of all intra-participant differences. All analyses and plotting were performed using R version 4.0.5 [59].

## 5. Conclusions

We report the variability around seven oxidative stress and eight inflammation markers in participants with GD1 and healthy controls. This information can be utilized for selecting outcome measures, calculations of sample size, and power analysis for future clinical studies.

## Figures and Tables

**Table 1:** Participant demographics.

Characteristic	Healthy Control (N = 18)	Gaucher Disease Type 1 (GD1)	
		GD-Naïve (N = 5)	GD-Treated (N = 13)
Female	9 (50%)	3 (60.0%)	8 (61.5%)
Caucasian	12 (75.0%)	5 (100%)	12 (92.3%)
Age (in years)	40.8 (15.3)	60.0 (9.3)	46.9 (12.0)
GD1 biomarkers			
CHITO (nmoles/h/mL)	<78.5 *	3590 (4182.5) ^1^	220 (224.2) ^7^
ACE (IU/L)	32.8–107.9 *	95.6 (86.7) ^2^	62.8 (35.2) ^5^
TRAP (IU/L)	0.28–9.84 *	25.2 (11.7) ^3^	7.18 (2.6) ^8^
Complete blood count			
Hemoglobin (g/L)	ND	13.6 (1.7)	14.4 (1.7) ^2^
Hematocrit (%)		39.4 (5.6) ^1^	42.3 (4.9) ^2^
Neutrophil (%)		72.5 (0.7) ^3^	55.2 (7.4) ^6^
Lymphocyte (%)		23.3 (9.2) ^2^	34.0 (6.8) ^5^
Eosinophils (%)		1.5 (0.7) ^3^	2.83 (3.4) ^6^
WBC (×10^9^/L)		5.62 (1.0)	6.02 (2.5) ^3^
Platelets (×10^9^/L)		122 (28.1)	189 (65.5) ^3^
Mutational status			
N370S/N370S	NA	4 (80%)	5 (38.5%)
N370S/L444P			5 (38.5%)
N370S/unknown			1 (7.7%)
N370S/R463C			1 (7.7%)
Unknown		1 (20%)	1 (7.7%)
GD1 therapy			
Years on therapy	NA	NA	16.1 (8.3)
ERT	NA		6 (46.1%)
SRT	NA		7 (53.8%)

Values presented are mean (SD) or N (%) as indicated. Superscript denotes the number of missing values. * The values are reported from literature [24] and are presented as a range where applicable; NA, Not applicable; or ND, not available. Abbreviations: CHITO, chitotriosidase; ACE, angiotensin-converting enzyme; TRAP, tartrate-resistant acid phosphatase; ERT, enzyme replacement therapy; SRT, substrate reduction therapy.

**Table 2:** Variability expressed as %CV, tabulated either overall or within different groups.

Biomarker	%CV Overall	%CV Healthy Control	%CV GD-Naïve	%CV GD-Treated
Total GSH	25.5	23.2	17.2	23.6
Ratio GSH/GSSG	82.6	58.6	16.7	86.7
CAT Activity	74.0	65.9	17.4	38.4
SOD Activity	55.7	66.4	29.7	42.7
GPx Activity	33.3	32.2	31.9	30.9
Protein Carbonyl	162.3	143.9	104.7	63.6
MDA	51.1	46.3	25.6	42.6
IL1RA	81.5	41.6	38.0	66.0
IL-6	60.4	69.8	39.3	56.5
IL-8	76.3	38.1	36.5	82.2
MCP1	51.0	50.6	36.7	54.1
MIP-1a	164.7	81.3	205.9	173.1
MIP-1b	138.1	87.5	60.9	84.0
TNFa	85.9	113.7	52.7	67.1
IL-10	701.6 *	129.4	138.5	484.4 *

* When the data for one participant from the GD-treated group was omitted because of apparent outlying measurements, %CV overall, %CV GD-treated were 114.1 and 86.1, respectively.

**Table 3:** Summary statistics for each biomarker separated by different groups.

Biomarker	Healthy Control	GD-Naïve	GD-Treated
	N = 18	N = 5	N = 13
Total_GSH (µg/mL)			
Mean (SD)	620 (144)	516 (89)	483 (114)
Median (IQR)	614 (189)	509 (122)	439 (163)
Unknown	6	0	2
Ratio_GSHGSSG			
Mean (SD)	29 (17)	6 (1)	15 (13)
Median (IQR)	27 (28)	6 (2)	10 (15)
Unknown	5	0	2
CAT_Activity (nmol/min/mL/mg)			
Mean (SD)	563 (371)	321 (56)	245 (94)
Median (IQR)	388 (534)	326 (71)	244 (126)
Unknown	4	0	2
SOD_Activity (U/mL/mg)			
Mean (SD)	6.01 (3.99)	4.71 (1.40)	6.18 (2.64)
Median (IQR)	4.95 (2.69)	4.43 (1.36)	5.68 (4.11)
Unknown	4	0	2
GPx_Activity (nmol/min/mL/mg)			
Mean (SD)	18.0 (5.8)	14.1 (4.5)	15.2 (4.7)
Median (IQR)	19.2 (7.8)	14.4 (5.7)	15.0 (5.4)
Unknown	4	0	2
Protein_Carbonyl (nmol/mg)			
Mean (SD)	1.55 (2.23)	0.43 (0.45)	0.55 (0.35)
Median (IQR)	0.64 (0.80)	0.27 (0.33)	0.54 (0.39)
Unknown	4	0	1
MDA (nM/mg)			
Mean (SD)	41 (19)	90 (23)	68 (29)
Median (IQR)	38 (23)	88 (17)	65 (45)
Unknown	4	1	1
IL1RA (pg/mL)			
Mean (SD)	255 (106)	1043 (396)	694 (458)
Median (IQR)	256 (155)	1000 (513)	619 (540)
Unknown	5	0	1
IL-6 (pg/mL)			
Mean (SD)	1.82 (1.27)	2.14 (0.84)	2.30 (1.30)
Median (IQR)	2.29 (2.25)	2.08 (0.95)	2.26 (1.53)
Unknown	5	0	1
IL-8 (pg/mL)			
Mean (SD)	2.52 (0.96)	6.94 (2.53)	4.65 (3.82)
Median (IQR)	2.56 (0.90)	7.05 (2.35)	3.13 (2.52)
Unknown	5	0	1
MCP1 (pg/mL)			
Mean (SD)	81 (41)	90 (33)	98 (53)
Median (IQR)	68 (43)	89 (32)	78 (62)
Minimum–Maximum	32–219	31–139	45–304
Unknown	5	0	1
MIP-1a (pg/mL)			
Mean (SD)	16 (13)	17 (35)	26 (45)
Median (IQR)	15 (22)	0 (0)	1 (36)
Minimum–Maximum	0–43	0–102	0–194
Unknown	5	0	1
MIP-1b (pg/mL)			
Mean (SD)	8 (7)	87 (53)	25 (21)
Median (IQR)	7 (11)	71 (51)	22 (22)
Unknown	5	0	1
TNFa (pg/mL)			
Mean (SD)	0.73 (0.83)	1.86 (0.98)	1.40 (0.94)
Median (IQR)	0.27 (1.27)	1.84 (1.33)	1.58 (1.35)
Unknown	5	0	1
IL-10 (U/mL)			
Mean (SD)	0.34 (0.44)	0.39 (0.54)	4.42 (21.41) *
Median (IQR)	0.08 (0.91)	0.08 (1.00)	1.02 (1.17) *
Unknown	5	0	1

* When data for one participant from the GD-treated group was omitted, because of apparent outlying measurements, the mean (SD) and median (IQR) were 0.72 (0.62) and 0.94 (1.14), respectively.

## Data Availability

The data used for this analysis can be shared through a reasonable request to the corresponding author R.V.K., at rvkartha@umn.edu.

## References

[1] Meikle P.J. (1999). Prevalence of Lysosomal Storage Disorders. JAMA.

[2] Brady R.O., Kanfer J.N., Bradley R.M., Shapiro D. (1966). Demonstration of a deficiency of glucocerebroside-cleaving enzyme in Gaucher’s disease. J. Clin. Investig..

[3] Avenali M., Blandini F., Cerri S. (2020). Glucocerebrosidase Defects as a Major Risk Factor for Parkinson’s Disease. Front. Aging Neurosci..

[4] Maor G., Rencus-Lazar S., Filocamo M., Steller H., Segal D., Horowitz M. (2013). Unfolded protein response in Gaucher disease: From human to Drosophila. Orphanet J. Rare Dis..

[5] Lee R.E. (1982). The pathology of Gaucher disease. Prog. Clin. Biol. Res..

[6] Gegg M.E., Schapira A.H.V. (2016). Mitochondrial dysfunction associated with glucocerebrosidase deficiency. Neurobiol. Dis..

[7] Araujo M.E.G., Liebscher G., Hess M.W., Huber L.A. (2020). Lysosomal size matters. Traffic.

[8] Tantawy A.A.G. (2015). Cytokines in Gaucher disease: Role in the pathogenesis of bone and pulmonary disease. Egypt. J. Med. Hum. Genet..

[9] Halliwell B. (1994). Free radicals, antioxidants, and human disease: Curiosity, cause, or consequence?. Lancet.

[10] Roversi F.M., Galdieri L.C., Grego B.H.C., Souza F.G., Micheletti C., Martins A.M., D’Almeida V. (2006). Blood oxidative stress markers in Gaucher disease patients. Clin. Chim. Acta.

[11] Deganuto M., Pittis M.G., Pines A., Dominissini S., Kelley M.R., Garcia R., Quadrifoglio F., Bembi B., Tell G. (2007). Altered intracellular redox status in Gaucher disease fibroblasts and impairment of adaptive response against oxidative stress. J. Cell. Physiol..

[12] Cleeter M.W.J., Chau K.-Y., Gluck C., Mehta A., Hughes D.A., Duchen M., Wood N.W., Hardy J., Mark Cooper J., Schapira A.H. (2013). Glucocerebrosidase inhibition causes mitochondrial dysfunction and free radical damage. Neurochem. Int..

[13] Belmatoug N., Di Rocco M., Fraga C., Giraldo P., Hughes D., Lukina E., Maison-Blanche P., Merkel M., Niederau C., Plöckinger U. (2017). Management and monitoring recommendations for the use of eliglustat in adults with type 1 Gaucher disease in Europe. Eur. J. Intern. Med..

[14] Balwani M., Burrow T.A., Charrow J., Goker-Alpan O., Kaplan P., Kishnani P.S., Mistry P., Ruskin J., Weinreb N. (2016). Recommendations for the use of eliglustat in the treatment of adults with Gaucher disease type 1 in the United States. Mol. Genet. Metab..

[15] Hayes R.P., Grinzaid K.A., Duffey E.B., Elsas II L.J. (1998). The impact of Gaucher disease and its treatment on quality of life. Qual. Life Res..

[16] Chen Zion Y., Pappadopulos E., Wajnrajch M., Rosenbaum H. (2016). Rethinking fatigue in Gaucher disease. Orphanet J. Rare Dis..

[17] Kartha R.V., Terluk M.R., Brown R., Travis A., Mishra U.R., Rudser K., Lau H., Jarnes J.R., Cloyd J.C., Weinreb N.J. (2020). Patients with Gaucher disease display systemic oxidative stress dependent on therapy status. Mol. Genet. Metab. Rep..

[18] Shemesh E., Deroma L., Bembi B., Deegan P., Hollak C., Weinreb N.J., Cox T.M., Shemesh E. (2013). Enzyme replacement and substrate reduction therapy for Gaucher disease. Cochrane Database of Systematic Reviews.

[19] (2014). FDA Center for Drug Evaluation and Research, Medical Review [CERDELGA]. https://www.accessdata.fda.gov/drugsatfda_docs/nda/2014/205494Orig1s000MedR.pdf.

[20] Mistry P.K., Lukina E., Ben Turkia H., Amato D., Baris H., Dasouki M., Ghosn M., Mehta A., Packman S., Pastores G. (2015). Effect of Oral Eliglustat vs Placebo on Spleen Volume in Patients with Splenomegaly and Gaucher Disease Type 1: The ENGAGE Randomized Clinical Trial. JAMA.

[21] Mistry P.K., Lukina E., Ben Turkia H., Shankar S.P., Feldman H., Ghosn M., Mehta A., Packman S., Lau H., Petakov M. (2021). Clinical outcomes after 4.5 years of eliglustat therapy for Gaucher disease type 1: Phase 3 ENGAGE trial final results. Am. J. Hematol..

[22] Pastores G.M., Weinreb N.J., Aerts H., Andria G., Cox T.M., Giralt M., Grabowski G.A., Mistry P.K., Tylki-Szymańska A. (2004). Therapeutic goals in the treatment of Gaucher disease. Semin. Hematol..

[23] Barak V., Acker M., Nisman B., Kalickman I., Abrahamov A., Zimran A., Yatziv S. (1999). Cytokines in Gaucher’s disease. Eur. Cytokine Netw..

[24] Seattle Children’s Hospital Gaucher Disease Biomarker Panel. https://seattlechildrenslab.testcatalog.org/show/LAB3073-1.

[25] Gervas-Arruga J., Cebolla J.J., de Blas I., Roca M., Pocovi M., Giraldo P. (2015). The Influence of Genetic Variability and Proinflammatory Status on the Development of Bone Disease in Patients with Gaucher Disease. PLoS ONE.

[26] Nair S., Branagan A.R., Liu J., Boddupalli C.S., Mistry P.K., Dhodapkar M.V. (2016). Clonal Immunoglobulin against Lysolipids in the Origin of Myeloma. N. Engl. J. Med..

[27] Pandey M.K., Grabowski G.A. (2013). Immunological Cells and Functions in Gaucher Disease. Crit. Rev. Oncog..

[28] Jmoudiak M., Futerman A.H. (2005). Gaucher disease: Pathological mechanisms and modern management. Br. J. Haematol..

[29] De Fost M., Out T.A., de Wilde F.A., Tjin E.P.M., Pals S.T., van Oers M.H.J., Boot R.G., Aerts J.F.M.G., Maas M., vom Dahl S. (2008). Immunoglobulin and free light chain abnormalities in Gaucher disease type I: Data from an adult cohort of 63 patients and review of the literature. Ann. Hematol..

[30] Allen M.J., Myer B.J., Khokher A.M., Rushton N., Cox T.M. (1997). Pro-inflammatory cytokines and the pathogenesis of Gaucher’s disease: Increased release of interleukin-6 and interleukin-10. QJM.

[31] Hollak C.E.M., Evers L., Aerts J.M.F.G., van Oers M.H.J. (1997). Elevated Levels of M-CSF, sCD14 and IL8 in Type 1 Gaucher Disease. Blood Cells Mol. Dis..

[32] Van Breemen M.J., de Fost M., Voerman J.S.A., Laman J.D., Boot R.G., Maas M., Hollak C.E.M., Aerts J.M., Rezaee F. (2007). Increased plasma macrophage inflammatory protein (MIP)-1α and MIP-1β levels in type 1 Gaucher disease. Biochim. Biophys. Acta Mol. Basis Dis..

[33] Mello A.S., da Silva Garcia C., de Souza Machado F., da Silva Medeiros N., Wohlenberg M.F., Marinho J.P., Dani C., Funchal C., Coelho J.C. (2015). Oxidative stress parameters of Gaucher disease type I patients. Mol. Genet. Metab. Rep..

[34] Zahran A.M., Elsayh K.I., El-Deek S.E.M., El-Baz M.A.H. (2015). Oxidative Stress, Trace Elements, and Circulating Microparticles in Patients with Gaucher Disease Before and After Enzyme Replacement Therapy. Clin. Appl. Thromb..

[35] Mozafari H., Khatami S., Kiani A., Rahimi Z., Vaisi-Raygani A., Afsharnaderi A., Alaei M.R. (2020). Oxidative Stress Parameters, Trace Elements, and Lipid Profile in Iranian Patients with Gaucher Disease. Biol. Trace Elem. Res..

[36] SRT in Comparison to ERT on Immune Aspects and Bone Involvement in Gaucher Disease [NCT02605603]. NCT02605603.

[37] Immune Biomarkers Related to Bone Pathology in Patients with Type 1 Gaucher Disease [NCT04055831]. NCT04055831.

[38] Trials and Research: Gaucher Online Disease Platform. https://gaucherdiseaseplatform.org/trials-and-research/.

[39] Oxidative Stress and Inflammatory Biomarkers in Gaucher Disease [NCT02437396]. NCT02437396.

[40] Altman D.G., Bland J.M. (1983). Measurement in Medicine: The Analysis of Method Comparison Studies. Stat..

[41] Martin Bland J., Altman D. (1986). Statistical Methods for Assessing Agreement Between Two Methods of Clinical Measurement. Lancet.

[42] Bland J.M., Altman D.G. (1995). Statistics notes: Calculating correlation coefficients with repeated observations: Part 1-correlation within subjects. BMJ.

[43] Schirmer M., Kumar V., Netea M.G., Xavier R.J. (2018). The causes and consequences of variation in human cytokine production in health. Curr. Opin. Immunol..

[44] Dabrosin C., Öllinger K., Ungerstedt U., Hammar M. (1997). Variability of Glutathione Levels in Normal Breast Tissue and Subcutaneous Fat during the Menstrual Cycle: An in Vivo Study with Microdialysis Technique. J. Clin. Endocrinol. Metab..

[45] Blanco R.A., Ziegler T.R., Carlson B.A., Cheng P.-Y., Park Y., Cotsonis G.A., Accardi C.J., Jones D.P. (2007). Diurnal variation in glutathione and cysteine redox states in human plasma. Am. J. Clin. Nutr..

[46] Woods J.A., Vieira V.J., Keylock K.T. (2006). Exercise, Inflammation, and Innate Immunity. Neurol. Clin..

[47] Suzuki K., Nakaji S., Yamada M., Totsuka M., Sato K., Sugawara K. (2002). Systemic inflammatory response to exhaustive exercise. Cytokine kinetics. Exerc. Immunol. Rev..

[48] Elokda A.S., Nielsen D.H. (2007). Effects of exercise training on the glutathione antioxidant system. Eur. J. Cardiovasc. Prev. Rehabil..

[49] De Jager W., Bourcier K., Rijkers G.T., Prakken B.J., Seyfert-Margolis V. (2009). Prerequisites for cytokine measurements in clinical trials with multiplex immunoassays. BMC Immunol..

[50] Petrovsky N., McNair P., Harrison L.C. (1998). Diurnal Rhythms of Pro-Inflammatory Cytokines: Regulation by Plasma Cortisol and Therapeutic Implications. Cytokine.

[51] Petrovsky N., Harrison L.C. (1998). The Chronobiology of Human Cytokine Production. Int. Rev. Immunol..

[52] Smaaland R., Sothern R.B., Laerum O.D., Abrahamsen J.F. (2002). Rhythms in human bone marrow and blood cells. Chronobiol. Int..

[53] Chiolero A., Paradis G., Rich B., Hanley J.A. (2013). Assessing the Relationship between the Baseline Value of a Continuous Variable and Subsequent Change Over Time. Front. Public Health.

[54] Food and Drug Administration (2015). Analytical Procedures and Methods Validation for Drugs and Biologics Guidance for Industry. https://www.fda.gov/media/87801/download.

[55] Knight V., Long T., Meng Q.H., Linden M.A., Rhoads D.D. (2020). Variability in the Laboratory Measurement of Cytokines. Arch. Pathol. Lab. Med..

[56] Rudež G., Meijer P., Spronk H.M.H., Leebeek F.W.G., Ten Cate H., Kluft C., de Maat M.P.M. (2009). Biological variation in inflammatory and hemostatic markers. J. Thromb. Haemost..

[57] Meijers W.C., van der Velde A.R., Muller Kobold A.C., Dijck-Brouwer J., Wu A.H., Jaffe A., de Boer R.A. (2017). Variability of biomarkers in patients with chronic heart failure and healthy controls. Eur. J. Heart Fail..

[58] Mallard A.R., Hollekim-Strand S.M., Ingul C.B., Coombes J.S. (2020). High day-to-day and diurnal variability of oxidative stress and inflammation biomarkers in people with type 2 diabetes mellitus and healthy individuals. Redox Rep..

[59] R Core Team (2021). R: A Language and Environment for Statistical Computing.

